# Association Between Postpartum Breast Cancer Diagnosis and Metastasis and the Clinical Features Underlying Risk

**DOI:** 10.1001/jamanetworkopen.2018.6997

**Published:** 2019-01-11

**Authors:** Erica T. Goddard, Solange Bassale, Troy Schedin, Sonali Jindal, Jeremy Johnston, Ethan Cabral, Emile Latour, Traci R. Lyons, Motomi Mori, Pepper J. Schedin, Virginia F. Borges

**Affiliations:** 1 Public Health Sciences Division, Fred Hutchinson Cancer Research Center, Seattle, Washington; 2 Translational Research Program and Human Biology Division, Fred Hutchinson Cancer Research Center, Seattle, Washington; 3 Knight Cancer Institute, Oregon Health & Science University, Portland; 4 Young Women’s Breast Cancer Translational Program, University of Colorado Anschutz Medical Campus, Aurora; 5 Division of Medical Oncology, Department of Medicine, University of Colorado Anschutz Medical Campus, Aurora; 6 Department of Cell, Developmental and Cancer Biology, Oregon Health & Science University, Portland; 7 University of Colorado Cancer Center, Aurora; 8 School of Public Health, Department of Medical Informatics & Clinical Epidemiology, School of Medicine, Biostatistics Shared Resource, Knight Cancer Institute, Oregon Health & Science University, Portland

## Abstract

**Importance:**

In women 45 years or younger, breast cancer diagnosis after childbirth increases the risk for metastasis and death, yet limited data exist to define this window of risk and associated prognostic factors.

**Objective:**

To assess the window of elevated risk for metastasis following a postpartum breast cancer (PPBC) diagnosis and whether clinical prognostic factors are associated with the increased risk.

**Design, Setting, and Participants:**

This multicenter cohort study conducted using cases from the Colorado Young Women’s Breast Cancer Cohort diagnosed between January 1, 1981, and December 31, 2014, included 701 women 45 years or younger with stage I to III invasive breast cancer for whom parity data, including time of last childbirth, were available. Data analysis was conducted from July 1 to September 30, 2017. This study involved a tertiary care academic hospital–based breast center and its regional affiliates with cases from the greater Rocky Mountain region.

**Exposures:**

Primary exposures were prior childbirth or no childbirth, time between most recent childbirth and breast cancer diagnosis, and time between breast cancer diagnosis and metastasis.

**Main Outcomes and Measures:**

The primary outcome was distant metastasis–free survival.

**Results:**

A total of 701 women 45 years or younger from the greater Rocky Mountain states region were included in the analysis; mean (SD) age at diagnosis was 37.9 (5.1) years. Breast cancer diagnosis within 10 years after parturition was associated with elevated risk for metastasis, particularly in women with stage I or II disease. In addition, women with PPBC diagnosed within 10 years of a completed pregnancy that was estrogen receptor–positive showed distant metastasis–free survival similar to that of nulliparous patients with estrogen receptor–negative cancer, and women with estrogen receptor–negative PPBC had further reduced metastasis-free survival. Moreover, women with PPBC had increased lymphovascular invasion and lymph node involvement. In addition, tumor-associated Ki67 positivity identified 129 patients with luminal B cancer in the cohort that, independent of parity status, had poorer prognosis compared with patients with luminal A cancer, although it did not reach statistical significance.

**Conclusions and Relevance:**

Diagnosis of PPBC within 10 years post partum appears to be associated with an increased risk for metastasis. This increased risk was highest in stages I and II cancer at diagnosis and present in both patients with estrogen receptor–positive and estrogen receptor–negative cancer, persisting in estrogen receptor–positive cases for up to 15 years after diagnosis. Postpartum breast cancer diagnoses were not associated with increased Ki67 index but were associated with increased lymphovascular invasion and lymph node involvement compared with breast cancer in nulliparous patients.

## Key Points

**Question:**

Is there an increased risk for metastasis of breast cancers that are diagnosed in young women post partum that extends beyond 5 years from the last childbirth, and what association do standard clinical prognostic factors have with metastatic risk in these young women when categorized by parity?

**Findings:**

In a cohort study of 701 women 45 years or younger with breast cancer, those with stage I or II cancer diagnosed up to 10 years post partum had an increased risk for distant metastasis, with both estrogen receptor–positive and estrogen receptor–negative disease significantly affected.

**Meaning:**

Postpartum status may be a prognostic indicator in young women with breast cancer and should be routinely identified, as up to 45% of women 45 years or younger with breast cancer fall into this category and could be at increased risk for metastasis.

## Teaser

This cohort study evaluates the risk of metastasis following a postpartum breast cancer diagnosis and clinical factors associated with this risk in women 45 years or younger.

## Introduction

Postpartum breast cancer (PPBC), previously defined as a breast cancer diagnosis within 5 years of last childbirth, has an approximately 3-fold increased risk for metastasis and death.^1,2,3^ This risk is specific to patients with cancer diagnosed post partum, as women with cancer diagnosed during pregnancy have a prognosis comparable to that of nulliparous patients.^1,4^ The high risk for metastasis is independent of poor prognostic indicators, including biological subtype, stage, age, or year of diagnosis.^1,2,3,5,6,7^ Yet, PPBC is an underrecognized subset of breast cancer, and few studies address the associated high risk for metastasis.^8^ As a result, strategies are lacking to identify young women with breast cancer who are at greatest risk and to develop targeted treatment options.^8^

Involution of the breast is a biological event that occurs post partum and may explain the increased risk for PPBC metastasis. After lactation or following childbirth in the absence of lactation, the breast undergoes a cell-death–mediated process characterized by robust tissue remodeling.^9,10^ During this physiologic event, the rodent mammary gland displays increased immune cell influx,^11,12,13^ remodeling of the extracellular matrix,^14,15^ and lymphangiogenesis^16^—events consistent with wound healing. In rodent models, postpartum involution is sufficient to increase tumor invasion and metastasis.^15,16,17^ From these observations the involution hypothesis was proposed, stating that the physiologically regulated wound-healing process of involution increases metastatic efficiency.^18^ In support of this hypothesis, the human postpartum breast also exhibits immune cell influx,^11,19^ a wound-healing extracellular matrix pattern, and lymphangiogenesis.^16^ In addition, in 1 small study, PPBC diagnosed within 2 years of childbirth had increased lymph node (LN) involvement independent of tumor size.^16^ Given the data implicating postpartum involution in promoting PPBC metastasis, it is important to define the duration of the window for increased metastatic risk as well as the contribution of known clinical attributes.

To evaluate whether the risk for increased metastasis of PPBC extends to patients diagnosed beyond 5 years after parturition and to investigate whether increased tumor cell proliferation, increased lymphovascular invasion (LVI), LN involvement, or other clinical attributes of PPBC correlate with metastatic risk in this patient population. To this end, we conducted a cohort study using the Colorado Young Women’s Breast Cancer Cohort, comprising women with breast cancer diagnosed at age 45 years or younger with extensive clinical and parity data.

## Methods

### Study Population

This study included women from the Colorado Young Women’s Breast Cancer Cohort who were prospectively recruited between January 1, 2004, and December 31, 2014, or retrospectively identified from January 1, 1981, to December 31, 2004. Women were eligible if they were 45 years or younger at diagnosis, had complete parity data, had completed follow-up for distant metastasis, and had received definitive tumor treatment with curative intent (N = 802).

The study was performed in compliance with institutional review board approval from the 2 participating institutions: the University of Colorado Anschutz Medical Center, Aurora, which is a tertiary-care, academic institution with a wide referral base from the greater Rocky Mountain area, and its affiliate, Vail Health Hospital, Vail, Colorado, which is a rural, community-based cancer center. Women in the prospective trial from 2004 to 2014 provided written informed consent. Those in the retrospective part of the study from 1981 to 2004 were Health Insurance Portability and Accountability Act and consent exempt with institutional review board approval. There was no financial compensation. This study followed the Strengthening the Reporting of Observational Studies in Epidemiology (STROBE) reporting guideline.

Distant metastases were defined as the identification of metastasis to organs outside of the ipsilateral breast and local draining LNs following a primary breast cancer diagnosis determined by radiologic imaging, physical examination, and/or pathological confirmation. Participants with no evidence of a distant recurrence at the end of follow-up were censored at the date of last contact. Participants with a diagnosis of stage IV cancer, missing data on stage of cancer or year of diagnosis, or who were pregnant at the time of diagnosis were excluded from the distant metastasis–free analysis (except as shown in eFigure 1 in the Supplement), resulting in 701 participants for the primary young women’s breast cancer analysis. For the analyses categorized by estrogen receptor (ER) status, cases with no hormone receptor data were excluded, resulting in 515 participants. For Ki67 analyses, participants with tissue available (n = 351) were initially included; of these, women with unknown tumor size or year of diagnosis were excluded, resulting in 272 participants.

Nulliparous patients, defined as women who never had a completed childbirth (n = 217), were evaluated. Nulliparous women with an incomplete pregnancy were included (62 of 217 [28.6%] of all nulliparous patients) because an intended preanalysis of the data suggested no association between an incomplete pregnancy and distant metastasis–free survival (MFS) (eFigure 2 in the Supplement). Participants were identified as having an incomplete pregnancy if their gynecologic history was GXP0, where *X* could be any number, representing prior pregnancy (gravidity [G]) of *X* number with no prior childbirth (parity [P]). Information on duration of pregnancy before loss and method of loss (spontaneous, therapeutic, or later trimester miscarriage) was not routinely available.

Parous patients were defined as those with cancer based on time from last birth to diagnosis and categorized as PPBC diagnosed within 5 years (n = 175), in 5 to less than 10 years (n = 153), or in 10 years or more (n = 156) following parturition. For some analyses, PPBC diagnosed within 5 years and in 5 to less than 10 years were combined into a single group of PPBC less than 10 years (n = 328) because risk for metastasis is similarly high in both groups and to increase sample sizes for statistical analyses. Mean follow-up was 5.6 years (range, 0-29.8 years). Patients with no evidence of distant MFS at the end of follow-up were censored at the time of last contact.

### End Points

The primary end point of analysis was distant MFS, defined as the duration of time between diagnosis and detection of metastatic disease or death, whichever occurred first. Because death without metastasis was rare in this patient population, distant metastasis accounted for almost all of the events of interest. Distant metastasis was considered present if definitive evidence for metastasis to organs beyond the ipsilateral breast and local draining LN was identified by radiologic imaging, physical examination, and/or pathological test results confirmation after a primary breast cancer diagnosis.

 Predetermined secondary end points included distant MFS in parity subtypes as influenced by stage, tumor size, LN involvement, LVI, tumor biological subtype, and Ki67-labeling index. For Ki67 analysis, tumor tissue was collected from the breast primary site at definitive surgery or by core biopsy before neoadjuvant chemotherapy. Lymph node status was determined by American Joint Committee on Cancer guidelines.^20^ Lymphovascular invasion was identified by pathological test report or study pathologist review of case slides. Follow-up data were obtained by medical record review and use of Colorado Tumor Registry data to confirm dates of metastasis, death, and last contact.

### Immunohistochemistry

Immunohistochemistry (IHC) staining for Ki67 (1:400; Clone SP6; Thermo Fisher Scientific) or pan-cytokeratin (1:500; Z0622; Dako) was performed as previously described.^15^ Positive control specimens (tonsil, spleen, and breast tissue) (eFigure 3A in the Supplement) and secondary-only negative controls were included in all IHC staining runs. To confirm that cases with less than 5% Ki67 staining did not have loss of antigenicity due to variable fixation protocols, patients without any prior positive IHC signal had antigenic integrity confirmed using pan-cytokeratin (eFigure 3B in the Supplement).

### Tissue Annotation and IHC Quantification

All IHC analyses were done blinded and overseen by a pathologist as previously described.^19^ A test set was used to confirm concordance between reviewers. Analysis of Ki67 was based on proposed guidelines by the International Ki-67 in Breast Cancer Working Group.^21^ Briefly, a minimum of 1000 tumor cells per tumor were annotated and assessed. A threshold signal of less than 14% or 14% or more Ki67 labeling index was used to assign low vs high thresholds and biological subtype to tumors as luminal A (low) and luminal B (high).

### Statistical Analysis

To test for intergroup differences, χ^2^, Fisher exact, 1-way analysis of variance, Kruskal-Wallis, or 2-way analysis of variance tests were used. The Kaplan-Meier method was applied to estimate the distant MFS probabilities. Log-rank tests were performed to assess differences between MFS probabilities across groups. Generalized additive models were used to evaluate the adequacy of PPBC categorization. Multivariable Cox proportional hazards regression analysis was performed to determine risk factors associated with MFS. Hazard ratios (HRs) and their 95% CIs were estimated. Factors accounted for in the overall analysis included biological subtype, stage, patient age, and year of diagnosis. The proportional hazard assumption was examined graphically using residual analyses proposed by Lin et al.^22^ For subsequent stage-categorized analyses, only biological subtype, age, and year of diagnosis were adjusted for.

For comparison of the nulliparous and PPBC less than 10 years groups categorized by ER status, we adjusted for stage, age, and year of diagnosis. For analyses stratified by Ki67 positivity, we adjusted for stage, age, and year of diagnosis. Adjusted survival curves were generated by estimating individual predictive probabilities using the Cox proportional hazards regression model and computing the predicted probabilities by parity group.

Patient data were collected and managed using REDCap. Statistical analyses were performed using Prism 7 software (GraphPad) or SAS, version 9.4 (SAS Institute Inc), and analyses were conducted from July 1 to September 30, 2017. A 2-sided *P* value less than .05 was considered statistically significant.

## Results

### Risk for Metastasis Analyzed by Parity Status

**Figure 1.**
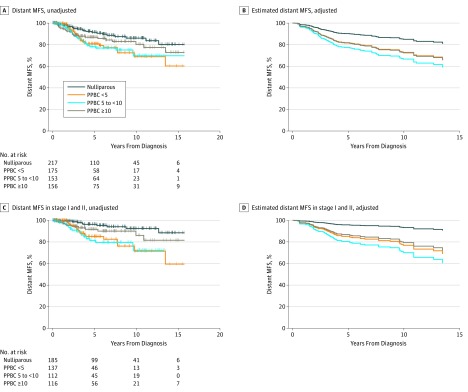
Risk for Metastasis in Young Women From the Colorado Young Women's Breast Cancer Cohort A, Unadjusted distant metastasis–free survival (MFS) (log rank test, *P* = .02) (eTable 1 in the Supplement). B, Distant MFS adjusted for stage, age, year of diagnosis, and tumor biological subtype. C, Unadjusted distant MFS in women with stage I or II cancer. D, Distant MFS in women with stage I or II cancer adjusted for age, year of diagnosis, and tumor biological subtype. PPBC <5 indicates postpartum breast cancer (PPBC) within 5 years of parturition; PPBC 5 to <10, PPBC within 5 to less than 10 years after parturition; and PPBC ≥10, PPBC after 10 or more years after parturition. Panels B and D show estimated values from adjusted Cox proportional hazards regression models; thus, including the number at risk is not appropriate.

The final analysis set included 701 participants with stages I, II, and III breast cancer in the Colorado Young Women’s Breast Cancer cohort; mean (SD) age was 37.9 (5.1) years. Clinical, demographic, and treatment data stratified by parity status are summarized in the Table. We first compared distant MFS across the nulliparous, PPBC less than 5 years, PPBC in 5 to less than 10 years, and PPBC after 10 or more years groups. Patients with PPBC less than 5 years and in 5 to less than 10 years exhibited more than a 2-fold increased metastasis risk compared with nulliparous patients (HR, 2.13; 95% CI, 1.21-3.74; *P* = .009 for PPBC <5 years; HR, 2.23; 95% CI, 1.26-3.93; *P* = .006 for PPBC 5 to <10 years, univariate analysis) (Figure 1A and eTable 1 in the Supplement). Patients with PPBC after 10 or more years had an intermediate risk for metastasis that did not reach statistical significance (HR, 1.6; 95% CI, 0.87-2.81; *P* = .13, univariate analysis) (Figure 1A and eTable 1 in the Supplement). Following adjustment for biological subtype, age, and year of diagnosis, the increased risk for metastasis in the PPBC less than 5 years group (HR, 1.53; 95% CI, 0.85-2.75; *P* = .16, Cox regression) and 5 to less than 10 years group (HR, 2.0; 95% CI, 1.10-3.59; *P* = .02, Cox proportional hazards regression) (Figure 1B and eTable 1 in the Supplement) no longer met statistical significance.

**Table.** University of Colorado Young Women's Breast Cancer Cohort

Characteristic	No. (%)				*P* Value
	Nulliparous (n = 217)	PPBC <5 y (n = 175)	PPBC 5 to <10 y (n = 153)	PPBC ≥10 y (n = 156)	
Age at diagnosis, mean (SD), y	36.9 (5.2)	35.5 (5.1)	38.5 (4.6)	41.3 (3.2)	<.001^a^
Biological subtype					
Luminal A (ER+, PR+, Her2−)	84 (38.7)	74 (42.3)	70 (45.8)	61 (39.1)	.66^b^
Luminal B (ER+, PR±, Her2+)	34 (15.7)	22 (12.6)	21 (13.7)	21 (13.5)	
Her2+ (ER−, PR−)	16 (7.4)	15 (8.6)	13 (8.5)	11 (7.1)	
Triple negative (ER−, PR−, Her2−)	32 (14.7)	35 (20.0)	17 (11.1)	28 (17.9)	
Missing Her2	38 (17.5)	20 (11.4)	16 (10.5)	27 (17.3)	
Missing ER or PR	2 (0.9)	2 (1.1)	3 (2.0)	0	
Other	11 (5.1)	7 (4.0)	13 (8.5)	8 (5.1)	
Estrogen status					
ER+	142 (65.4)	114 (65.1)	104 (68.0)	97 (62.2)	.78^b^
ER−	62 (28.6)	53 (30.3)	40 (26.1)	53 (34.0)	
Missing	13 (5.6)	8 (4.6)	9 (5.9)	6 (3.8)	
Histologic grade					
Grade I	25 (11.5)	11 (6.3)	14 (9.2)	22 (14.1)	.08^b^
Grade II	70 (32.3)	51 (29.1)	55 (36.0)	61 (39.1)	
Grade III	104 (47.9)	96 (54.9)	73 (47.7)	57 (36.5)	
Missing	18 (8.3)	17 (9.7)	11 (7.2)	16 (10.3)	
Tumor size					
DCIS	0	1 (0.6)^c^	0	0	.42^b^
0.1 to ≤2.0 cm	110 (50.7)	85 (48.6)	63 (41.2)	75 (48.1)	
>2.0 to ≤5.0 cm	69 (31.8)	57 (32.6)	56 (36.6)	54 (34.6)	
>5.0 cm	17 (7.8)	17 (9.7)	19 (12.4)	20 (12.8)	
Missing	21 (9.7)	15 (8.6)	15 (9.8)	7 (4.5)	
Stage					
I	87 (40.1)	46 (26.3)	46 (30.1)	52 (33.3)	.01^b^
II	98 (45.2)	91 (52.0)	66 (43.1)	64 (41.0)	
III	32 (14.7)	38 (21.7)	41 (26.8)	40 (25.6)	
Year of diagnosis					
1980-1998	45 (20.7)	30 (17.1)	19 (12.4)	50 (32.1)	<.001^b^
1999-2004	62 (28.6)	32 (18.3)	48 (31.4)	40 (25.6)	
2005-2014	110 (50.7)	113 (64.6)	86 (56.2)	66 (42.3)	
BMI, mean (SD)	25.0 (6.9)	26.1 (6.0)	26.0 (6.0)	26.7 (7.0)	.03^a^
Chemotherapy					
Yes	136 (62.7)	132 (75.4)	100 (65.4)	93 (59.6)	.02^b^
No	38 (17.5)	15 (8.6)	25 (16.3)	22 (14.1)	
Missing	43 (19.8)	28 (16.0)	28 (18.3)	41 (26.3)	
Radiotherapy					
Yes	99 (45.6)	76 (43.4)	74 (48.4)	57 (36.5)	.39^b^
No	55 (25.3)	45 (25.7)	31 (20.3)	41 (26.3)	
Missing	63 (29.0)	54 (30.9)	48 (31.4)	58 (37.2)	
Patients with metastasis	22 (10.1)	29 (16.6)	28 (18.3)	24 (15.4)	.09^b^

Abbreviations: BMI, body mass index (calculated as weight in kilograms divided by height in meters squared); DCIS, ductal carcinoma in situ; ER, estrogen receptor; PPBC, postpartum breast cancer; PR, progesterone receptor; +, positive; −, negative; ±, positive or negative.

^a^ Kruskal-Wallis test.

^b^ χ^2^ Test.

^c^ Indicates that DCIS in breast and positive lymph nodes is stage II.

We next assessed distant MFS between parity groups categorized by stage (Figure 1C and D and eTable 1 in the Supplement). Analysis of patients with stages I and II cancer (n = 550) revealed that patients with PPBC less than 5 years and in 5 to less than 10 years were at significantly increased risk for metastasis compared with nulliparous patients, even after adjusting for biological subtype, age, and year of diagnosis (HR, 3.5; 95% CI, 1.56-7.89; *P* = .002; and HR, 5.2; 95% CI, 2.26-11.82; *P* < .001; Cox proportional hazards regression) (Figure 1D and eTable 1 in the Supplement). In contrast, patients with stage III cancer (n = 151) exhibited a high risk of metastasis regardless of parity status (*P* > .05, Cox proportional hazards regression for all comparisons), for example, 44 vs 58 metastatic recurrences in ER-positive, nulliparous women vs those with PPBC less than 10 years at 5 years follow-up (eTable 1 in the Supplement).

### Evaluation of Known Clinical Risk Factors in PPBC 

**Figure 2.**
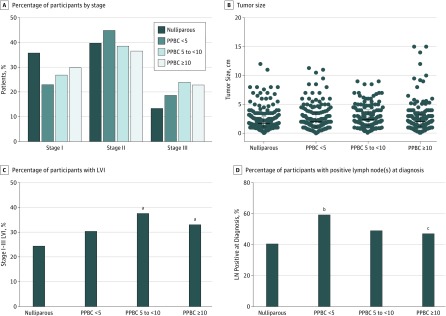
Lymphovascular Invasion (LVI) and Lymph Node (LN) Involvement Evaluated by Parity Groups A, Stage of disease at diagnosis in patients with stage I to III disease, categorized by parity group (2-way analysis of variance [ANOVA], Tukey multiple comparisons test); sample size is available in the Table. B, Tumor size at diagnosis categorized by parity group (1-way ANOVA, Tukey multiple comparisons test); error bars indicate median (interquartile range); sample size is available in the Table. C, Percentage of patients with stage I to III cancer presenting with LVI (χ^2^ test). Sample sizes were nulliparous: 101 LVI negative (LVI−), 53 LVI positive (LVI+), and 67 unknown; postpartum breast cancer (PPBC) within 5 years (PPBC <5): 73 LVI−, 53 LVI+, and 51 unknown; PPBC within 5 to less than 10 years (PPBC 5 to <10): 63 LVI−, 57 LVI+, and 33 unknown; PPBC after 10 years or more (PPBC ≥10): 76 LVI−, 51 LVI+, and 29 unknown. D, Percentage of patients with stage I to III cancer diagnosed with LN+ disease (χ^2^ test). Sample sizes were nulliparous, 130 LN− and 86 LN+; PPBC <5: 72 LN− and 102 LN+; PPBC 5 to <10: 78 LN− and 73 LN+; PPBC ≥10: 82 LN− and 71 LN+. ^a^*P* < .05 compared with nulliparous patients. ^b^*P* < .001 compared with nulliparous patients. ^c^*P* < .05 compared with the PPBC <5 group.

We next evaluated biological subtype, tumor stage, and tumor size across groups (Figure 2A and B and eFigure 4A and B in the Supplement) and observed no significant differences. For example, stage I and II breast cancer represented 85.3% of the nulliparous, 78.3% of the PPBC in less than 5 years, and 73.2% of the PPBC in 5 to 10 years cases. Thus, we evaluated frequency of LVI and LN involvement as potential explanations for increased metastasis in PPBC, as estimated from our preclinical models.^16^ Overall, the patients with PPBC in 5 to less than 10 years had higher LVI and the patients with PPBC less than 5 years group exhibited increased LN-positive disease at diagnosis (χ^2^ test) (Figure 2C and D). Consistent with increased LN-positive diagnoses, patients categorized as having PPBC less than 5 years received more chemotherapy than the nulliparous group (75.4% vs 62.7%, respectively; *P* = .007, χ^2^ test) (eFigure 4C in the Supplement and Table).

###  Metastatic Risk Assessed by ER and Parity Status

**Figure 3.**
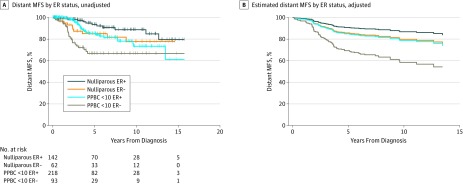
Analysis Between Parity and Estrogen Receptor (ER) Status for Association With Distant Metastasis–Free Survival (MFS) A, Proportions of distant MFS in the Colorado Young Women’s Breast Cancer Cohort categorized by parity and estrogen receptor status (log rank test, *P* < .001; eTable 2 in the Supplement). B, Cox proportional hazards regression model estimating proportions of distant MFS in the Colorado Young Women’s Breast Cancer Cohort categorized by parity and ER status adjusted for stage, age, and year of diagnosis. Because the curve is estimated, including the number of patients at risk is not appropriate. PPBC <10 indicates postpartum breast cancer (PPBC) diagnosed within 10 years.

Because breast tumor ER status is a prognostic indicator,^23,24^ we investigated the association of ER with distant MFS in PPBC and identified clear interactions. We found that patients with an ER-positive PPBC diagnosis exhibited distant MFS similar to that of nulliparous patients with ER-negative disease (HR, 1.02; 95% CI, 0.49-2.10) (Figure 3A and eTable 2 in the Supplement), with a 40% likelihood of metastasis by year 15. Furthermore, in women with ER-negative disease, a diagnosis of PPBC within 10 years of parturition increased the probability of developing metastatic disease by approximately 2-fold over nulliparous patients with ER-negative disease (HR, 2.19; 95% CI, 1.04-4.58) (Figure 3A and eTable 2 in the Supplement). Multivariable analyses adjusting for age and year of diagnosis confirmed these findings (Figure 3B and eTable 2 in the Supplement).

We also evaluated the combined outcome of parity and ER status on distant MFS categorized by stage. Nulliparous patients with stage I or II breast cancer had a low risk for metastasis regardless of ER status (eFigure 5A and eTable 2 in the Supplement). Patients with stages I and II PPBC diagnosed within 10 years of parturition exhibited a higher risk for distant metastasis (eFigure 5A and eTable 2 in the Supplement). In contrast, stage III distant MFS was not significantly different between the parity groups, although the patients with ER-negative cancer had a higher metastasis risk compared with those with ER-positive cancer (eFigure 5B and eTable 2 in the Supplement). Nulliparous and PPBC ER-negative groups exhibited 5-year metastasis-free survival rates of only approximately 50% compared with 75% in ER-positive cases regardless of parity status.

### Analysis of Tumor Proliferation Index by Parity Status

High Ki67 positivity is a biomarker used to estimate prognosis and distinguish ER-positive luminal A disease from ER-positive luminal B disease.^25,26,27^ Luminal subtype distinction is important, as patients with luminal A cancer have improved overall survival compared with those with luminal B cancer.^26,28,29^ To investigate potential interactions between Ki67 and parity status, we stained available primary tumor samples for Ki67 (eTable 3 and eFigure 3 in the Supplement). As estimated, Ki67-positive labeling index rose with increasing grade (mean [SEM], 1.73 [1.86] in grade 1 compared with 40.7 [1.76] in grade 3; *P* < .001) (eFigure 6A in the Supplement). We also observed a marginal elevation in Ki67 with increasing stage, but no significant difference in Ki67 levels when comparing tumor size, LVI, or LN involvement (eg, mean [SEM], 29.9 [1.76] in tumor size >0.1 to ≤2.0 cm compared with 34.3 [3.5] in tumor size >5 cm; *P* = .82) (eFigure 6B-E in the Supplement). There was no significant difference between patients with PPBC and nulliparous patients for Ki67 (mean [SEM], 34.3 [2.3] in PPBC less than 5 cases compared with 30.6 [2.3] in nulliparous patients; *P* = .30) (eFigure 6F in the Supplement). In addition, we observed reduced Ki67 with increasing age at diagnosis (mean [SEM], 37.5 [3.3] in patients ≤30 years old at diagnosis compared with 27.3 [1.8] in patients >40 to 45 years old at diagnosis; *P* = .03) (eFigure 6G in the Supplement), as previously described.^30,31^

### Updated Classification of Luminal Tumors Using Ki67 Staining Results

**Figure 4.**
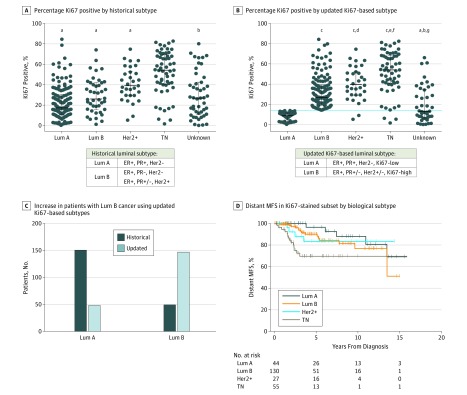
Incorporation of Ki67 Labeling Index for Updated Luminal Subtype Definitions A, Intratumoral Ki67 staining index by tumor biological subtype using historic luminal A (Lum A) and luminal B (Lum B) definitions. B, Ki67-based Lum A (Ki67 low) and Lum B (Ki67 high) definitions. Totals are 316 patients (A) and 320 patients (B) (1-way analysis of variance, Tukey multiple comparisons test; error bars indicate median and interquartile range). Estrogen receptor (ER), progesterone receptor (PR), and Her2 status were used in defining biological subtype. Triple negative (TN) indicates negative for all 3 markers. C, Number of patient tumors identified as Lum A and Lum B using historical and Ki67-based subtype definitions. D, Distant metastasis–free survival (MFS) categorized by biological subtype using Ki67-based definitions for Lum A and Lum B (log-rank test, *P* = .007; eTable 4 in the Supplement). Lum A, 44; Lum B, 130; Her2+, 27; TN, 55. + Indicates positive; −, negative; +/−, positive or negative. ^a^*P* < .001 vs TN. ^b^*P* < .001 vs Her2. ^c^*P* < .001 vs Lum A. ^d^*P* < .05 vs Lum B. ^e^*P* < .001 vs Lum B. ^f^*P* < .05 vs Her2*.* ^g^*P* < .01 vs Lum A.

Using one standard luminal B subtype definition (ER-positive, progesterone receptor [PR]–negative, Her2-negative or ER-positive, PR-positive or PR-negative, and Her2-positive) there was no significant difference in distant MFS between luminal A and B in our cohort (eFigure 7A and B in the Supplement). When a luminal B definition based on ER-positive, PR-positive or PR-negative, Her2-positive or Her2-negative, and greater than 14% Ki67-positive tumor cells^26^ was used, a large number of patients with the luminal A subtype shifted to the group with the luminal B subtype across all parity groups (Figure 4A-C and eFigure 7C in the Supplement), corroborating previous findings.^1,32^

We assessed distant MFS by biological subtype using the updated luminal classification and found that patients with luminal B cancers in this subset of our cohort do not have a statistically significantly increased risk for distant metastasis compared with those with luminal A cancers, even after adjusting for tumor size, age at diagnosis, and year of diagnosis (HR, 1.71; 95% CI, 0.56-5.28; *P* = .35, multivariable analysis) (Figure 4D and eFigure 7D and eTable 4 in the Supplement).

## Discussion

Using the Colorado Young Women’s Breast Cancer Cohort, we present findings suggesting that breast cancer diagnosed in women 45 years or younger within 10 years following childbirth serves as an independent adverse prognostic factor by conferring an approximate 2-fold increased risk for distant metastasis compared with the risk in nulliparous patients. Among cases diagnosed at stage I or II, the increased risk in postpartum women was 3.5- to 5.0-fold higher than in nulliparous women and remained significant after adjusting for known prognostic clinical features. Patients with stage III cancer exhibited a uniformly poor prognosis regardless of parity status. These data suggest that the window of risk for metastasis imparted by a postpartum diagnosis persists for longer than previously reported.^1^ Furthermore, the data highlight recent childbirth as an identifiable biomarker that may be useful for determining prognosis of young women’s breast cancers.

Extending the definition of patients with PPBC who are at high risk for distant metastasis to include patients diagnosed with breast cancer within 10 years of parturition suggests that patients with PPBC now compose approximately 45% of all young women’s breast cancers (eFigure 1 in the Supplement). Taking into account US annual statistics of 25 000 new breast cancer diagnoses in women 45 years or younger,^33,34,35^ just over 12 000 cases per year would meet the PPBC criterion for poor prognostic disease. Older age at first birth correlates with increased risk for PPBC^18,36,37^; with more women opting to delay childbearing,^38,39,40^ the incidence of PPBC is likely increasing.^8,41,42^

Despite these findings, PPBC remains an underrecognized subset of breast cancer.^8^ Moreover, these data suggest that stages I and II breast cancer in patients with PPBC diagnosed within 10 years of parturition may be underestimated in their risk for metastasis, as parity status is not currently factored into clinical decision-making algorithms, such as the National Comprehensive Cancer Network guidelines.^42^ In sum, we suggest that poor-prognostic PPBC is an increasing problem that merits more dedicated research.

Possible explanations for the increase in metastasis were evaluated herein, including increased tumor size at diagnosis (as a surrogate for delayed diagnosis) and clinical variables, such as enrichment of poor prognostic biological subtypes, LVI, and LN involvement. The age range of our patients is predominantly below the age at which guidelines recommend initiation of breast cancer screening; thus, most of these cases are detected based on clinical symptoms. We observed no difference in stage or tumor size between the PPBC and nulliparous groups, suggesting no bias toward delayed diagnosis in PPBC. Our results are concordant with other reports in which nulliparous women with breast cancer and those with PPBC had similarly sized tumors and staged disease.^5,6^

It is well documented that premenopausal women present with an increased frequency of triple-negative breast cancer compared with postmenopausal women.^43,44,45,46^ However, the frequencies of biological subtypes do not differ by parity status in other cohorts of young women with breast cancer.^1,32^ In a study using a large African American cohort, patients with premenopausal breast cancer had similar rates of ER-negative disease regardless of parity status.^47^ In the present study, biological subtype did not differ by parity status, but the risk for metastasis in patients diagnosed with PPBC within 10 years of parturition appeared to be compounded by an ER-negative diagnosis. Women with PPBC within 10 years of parturition with ER-positive disease had similar rates of metastasis compared with nulliparous patients with ER-negative disease. These data identify a postpartum diagnosis itself as an independent risk factor for metastasis, especially for early-stage disease. Furthermore, our data suggest that a postpartum diagnosis and ER-negative disease may be an additive component of risk for metastasis.

Proliferation index has garnered substantial attention as a prognostic indicator in breast cancer, as molecular subtyping of human breast cancers using Ki67 can differentiate good prognostic luminal A from poor prognostic luminal B subtypes.^48^ Our quantification of tumor Ki67 labeling revealed an increase in luminal B diagnoses in young women’s breast cancers. These data are consistent with published work showing a high rate of luminal B diagnoses in young women.^1,32^ However, in our cohort, Ki67 did not correlate with parity status, suggesting that, although Ki67 likely assists with identifying patients with ER-positive cancer at greatest risk for metastasis, it does not appear to delineate postpartum patients at increased risk. Furthermore, we conclude that enrichment of poor prognostic biological subtypes or highly proliferative primary tumors does not explain the increased risk for metastasis in patients with PPBC within 10 years of parturition. Instead, our data suggest that the postpartum period and the changes incurred by the mammary microenvironment during this life event may drive the increased metastasis.

Consistent with this hypothesis, studies of the postpartum mammary microenvironment performed in rodents and women implicate the wound healing–like program of postpartum involution in lymphangiogenesis, LVI, entry into the circulation, LN involvement, and increased risk for distant metastasis.^11,15,16,17,49,50^ Herein, our clinical data lend support for this theory, with a high frequency of patients with PPBC within 10 years of parturition presenting with LVI and/or LN involvement. Data also support a previous observation that elevated breast lymphatics may persist up to 10 years following a completed pregnancy^16^ and suggest that tumor cell interaction with the postpartum lymphatics influences their metastatic efficiency, as previously reported.^51,52^ However, a direct link between microenvironmental factors in postpartum patients and increased risk for metastasis has yet to be identified.

### Limitations

Our study is limited in part by demographics, as the patient population of women, primarily from Colorado, is largely non-Hispanic white, relatively lean, and with low breast cancer mortality rates.^53^ These features of our patient population could introduce a bias toward better outcomes with overall less metastasis than might be expected in a more diverse cohort. Another limitation of our study is the use of combined prospective and retrospective data collection; the retrospective cases are prone to missing data yet offer the longest follow-up.

## Conclusions

This study suggests that patients with a PPBC diagnosis within 10 years of the most recent completed pregnancy have an increased risk for development of metastatic disease in stage I and II breast cancers of both ER-positive and ER-negative subtypes. These data provide further evidence that a PPBC diagnosis may represent a unique subtype of cancer that requires distinct clinical and translational research initiatives. These observations should be confirmed in other cohorts of young women with breast cancer for determination of generalizability.

## Notes

Supplement. **eFigure 1.** Postpartum Breast Cancers Diagnosed Within 10 Years of a Completed Pregnancy Account for 45% of All Young Women’s Breast Cancers **eFigure 2.** Exclusion of Nulliparous Patients With Incomplete Pregnancies Does Not Impact Distant Metastasis-Free Survival **eFigure 3.** Patient Tissue Immunohistochemistry Staining Controls **eFigure 4.** Biologic Subtype and Tumor Size Are Not Altered by Parity Status of Young Women’s Breast Cancer Patients **eFigure 5.** Interactions Between Parity, Estrogen Receptor Status, and Stage Determine Distant Metastasis-Free Survival in Young Women’s Breast Cancer **eFigure 6.** Intratumoral Ki67 Staining Index Is Increased With Increasing Grade and Stage and Is Reduced With Increasing Age at Diagnosis **eFigure 7.** Distant MFS in the Colorado Young Women’s Breast Cancer Cohort by Luminal Subtype **eTable 1.** University of Colorado Metastasis-Free Survival Analysis **eTable 2.** University of Colorado Metastasis-Free Survival Analysis, Stratified by ER Status **eTable 3.** Ki67 Staining Cohort **eTable 4.** University of Colorado Metastasis-Free Survival Analysis, Stratified by Biologic Subtype
