# Supplementary material for: Association Between Postpartum Breast Cancer Diagnosis and Metastasis and the Clinical Features Underlying Risk
Source: JAMA Netw Open. 2019 Jan 11;2(1):e186997. doi: 10.1001/jamanetworkopen.2018.6997 (PMC6484560; doi:10.1001/jamanetworkopen.2018.6997)
Supplement: Supplement. — eFigure 1. Postpartum Breast Cancers Diagnosed Within 10 Years of a Completed Pregnancy Account for 45% of All Young Women’s Breast Cancers eFigure 2. Exclusion of Nulliparous Patients With Incomplete Pregnancies Does Not Impact Distant Metastasis-Free Survival eFigure 3. Patient Tissue Immunohistochemistry Staining Controls eFigure 4. Biologic Subtype and Tumor Size Are Not Altered by Parity Status of Young Women’s Breast Cancer Patients eFigure 5. Interactions Between Parity, Estrogen Receptor Status, and Stage Determine Distant Metastasis-Free Survival in Young Women’s Breast Cancer eFigure 6. Intratumoral Ki67 Staining Index Is Increased With Increasing Grade and Stage and Is Reduced With Increasing Age at Diagnosis eFigure 7. Distant MFS in the Colorado Young Women’s Breast Cancer Cohort by Luminal Subtype eTable 1. University of Colorado Metastasis-Free Survival Analysis eTable 2. University of Colorado Metastasis-Free Survival Analysis, Stratified by ER Status eTable 3. Ki67 Staining Cohort eTable 4. University of Colorado Metastasis-Free Survival Analysis, Stratified by Biologic Subtype [file jamanetwopen-2-e186997-s001.pdf]

Supplementary Online Content

Goddard ET, Bassale S, Schedin T, et al. Association between postpartum breast cancer diagnosis and metastasis and the clinical features underlying risk. *JAMA Netw Open*. 2019;2(1):e186997. doi:10.1001/jamanetworkopen.2018.6997

eFigure 1. Postpartum Breast Cancers Diagnosed Within 10 Years of a Completed Pregnancy Account for 45% of All Young Women's Breast Cancers

eFigure 2. Exclusion of Nulliparous Patients With Incomplete Pregnancies Does Not Impact Distant Metastasis-Free Survival

eFigure 3. Patient Tissue Immunohistochemistry Staining Controls

eFigure 4. Biologic Subtype and Tumor Size Are Not Altered by Parity Status of Young Women's Breast Cancer Patients

eFigure 5. Interactions Between Parity, Estrogen Receptor Status, and Stage Determine Distant Metastasis-Free Survival in Young Women's Breast Cancer

eFigure 6. Intratumoral Ki67 Staining Index Is Increased With Increasing Grade and Stage and Is Reduced With Increasing Age at Diagnosis

eFigure 7. Distant MFS in the Colorado Young Women's Breast Cancer Cohort by Luminal Subtype

eTable 1. University of Colorado Metastasis-Free Survival Analysis

eTable 2. University of Colorado Metastasis-Free Survival Analysis, Stratified by ER Status

eTable 3. Ki67 Staining Cohort

eTable 4. University of Colorado Metastasis-Free Survival Analysis, Stratified by Biologic Subtype

This supplementary material has been provided by the authors to give readers additional information about their work.

© 2019 Goddard ET et al. *JAMA Network Open.*

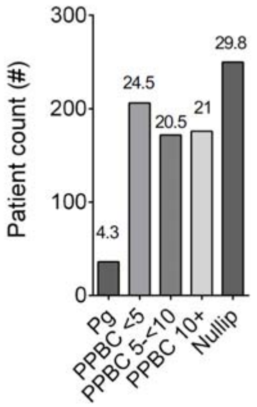

eFigure 1. Postpartum breast cancers diagnosed within 10 years of a completed pregnancy account for 45% of all young women's breast cancers. Patient counts by parity status, including the subset of patients diagnosed while pregnant (Pg). Patients diagnosed within 10 years of a pregnancy account for 45% of all patients in this cohort and pregnant patients account for 4.3%. Pg, n=36; PPBC<5, n=206; PPBC 5-<10, n=172; PPBC 10+, n=176; Nullip, n=250.

© 2019 Goddard ET et al. *JAMA Network Open.*

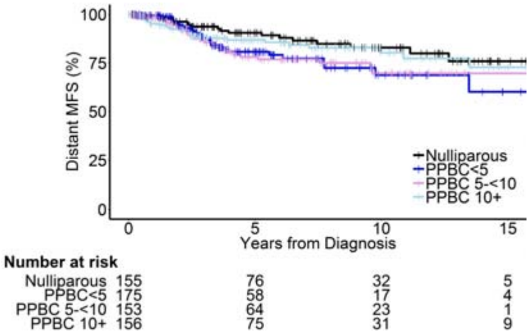

eFigure 2. Exclusion of nulliparous patients with incomplete pregnancies does not impact distant metastasis-free survival. Distant metastasis-free survival (MFS) in the Colorado Young Women's Breast Cancer Cohort in which nulliparous patients with incomplete pregnancies were excluded from analysis (Log Rank Test, P=0.02). Nullip, n=155; PPBC<5, n=175; PPBC 5-<10, n=153; PPBC 10+, n=156; Nullip with incomplete pregnancies excluded from analysis, n=62.

© 2019 Goddard ET et al. *JAMA Network Open.*

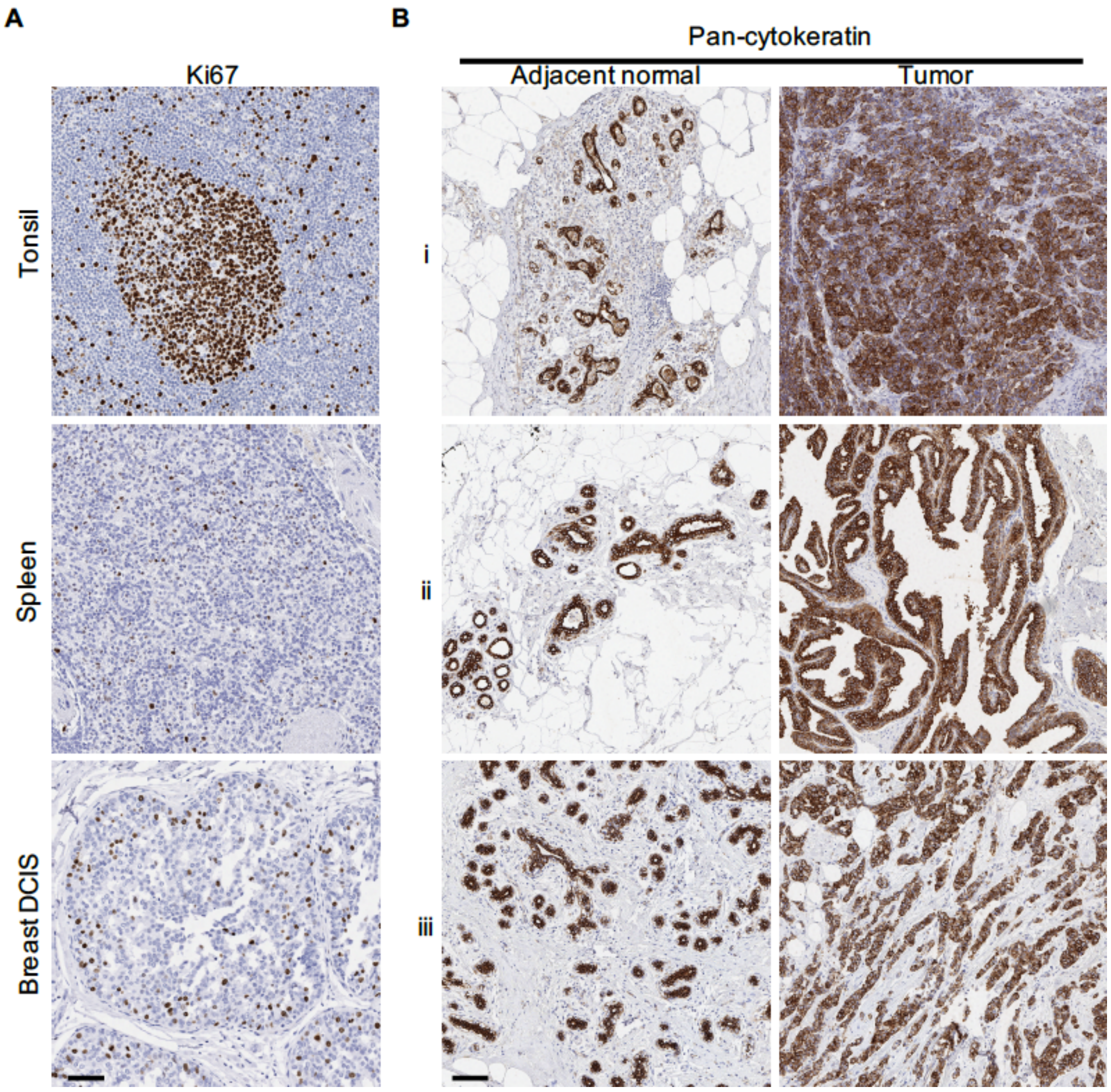

eFigure 3. Patient tissue immunohistochemistry staining controls. (A) Representative Ki67 staining in human tonsil (top), spleen (middle), and breast ductal carcinoma in situ (DCIS; bottom) served as positive controls on staining runs (scale bar = 100 μm) **(B)** Pan-cytokeratin staining to confirm antigenicity in three (i-iii) patient breast tumor samples that had low or negative antigen staining levels for numerous stains including Ki67. Representative pan-cytokeratin staining in adjacent normal breast tissue (left) and tumor (right) (scale bar = 300 μm).

© 2019 Goddard ET et al. *JAMA Network Open.*

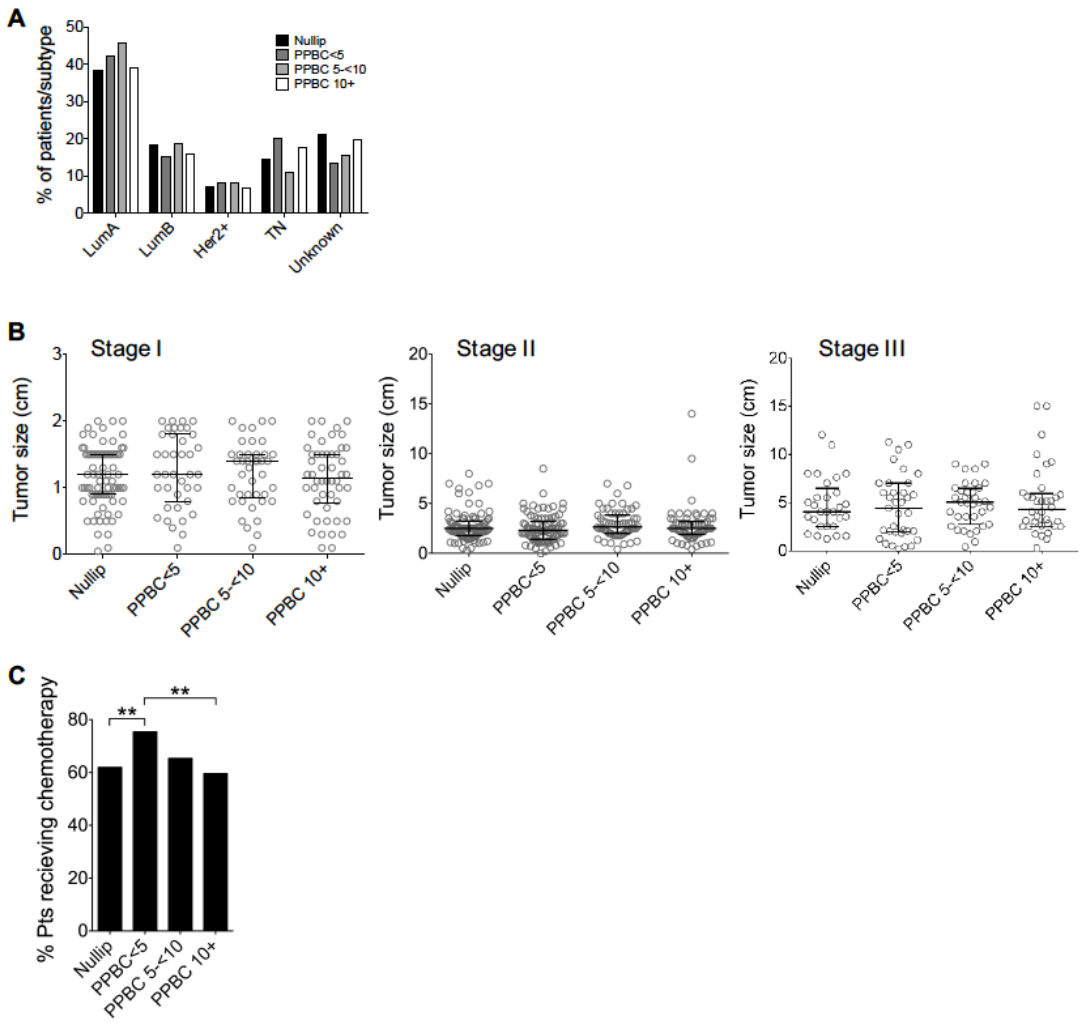

eFigure 4. Biologic subtype and tumor size are not altered by parity status of young women's breast cancer patients. (A) Biologic subtype at diagnosis in Stage I-III patients, categorized by parity group (two-way ANOVA, Tukey's multiple comparisons test). Luminal A (LumA): Nullip, n=85; PPBC<5, n=75; PPBC 5-<10, n=70; PPBC 10+, n=61. Luminal B (LumB): Nullip, n=41; PPBC<5, n=27; PPBC 5-<10, n=29; PPBC 10+, n=25. Her2+: Nullip, n=16; PPBC<5, n=15; PPBC 5-<10, n=13; PPBC 10+, n=11. TN (triple negative): Nullip, n=32; PPBC<5, n=36; PPBC 5-<10, n=17; PPBC 10+, n=28. Unknown: Nullip, n=47; PPBC<5, n=24; PPBC 5-<10, n=24; PPBC 10+, n=31. **(B)** Tumor size at diagnosis in stage I (left), stage II (middle), and stage III (right) patients (one-way ANOVA, Tukey's multiple comparisons test; error bars are median with interquartile range). **(C)** Percentage of Stage I-III patients treated with chemotherapy categorized by parity status (chi-squared test). Nullip: n=137 chemotherapy (chemo)+, n=40 chemo-, n=40 unknown; PPBC<5: n=134 chemo+, n=15 chemo-, n=28 unknown; PPBC 5-<10: n=100 chemo+, n=25 chemo-, n=28 unknown; PPBC 10+: n=93 chemo+, n=22 chemo-, n=41 unknown. **=p-value<0.01.

© 2019 Goddard ET et al. *JAMA Network Open.*

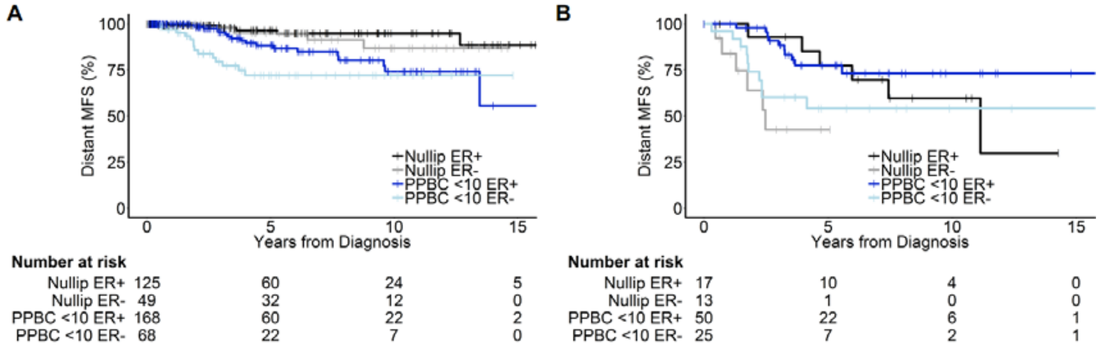

eFigure 5. Interactions between parity, estrogen receptor status, and stage determine distant metastasis-free survival in young women's breast cancer. (A) Distant metastasis-free survival (MFS) in stage I/II patients categorized by parity and estrogen receptor (ER) status. Nullip ER+, n=125; Nullip ER-, n=49; PPBC<10 ER+, n=168; PPBC<10 ER-, n=68 (Log Rank Test, P<0.0001, see eTable 2). **(B)** Distant MFS in stage III patients categorized by parity and ER status. Nullip ER+, n=17; Nullip ER-, n=13; PPBC<10 ER+, n=50; PPBC<10 ER-, n=25 (Log Rank Test, P=0.005, see eTable 2).

© 2019 Goddard ET et al. *JAMA Network Open.*

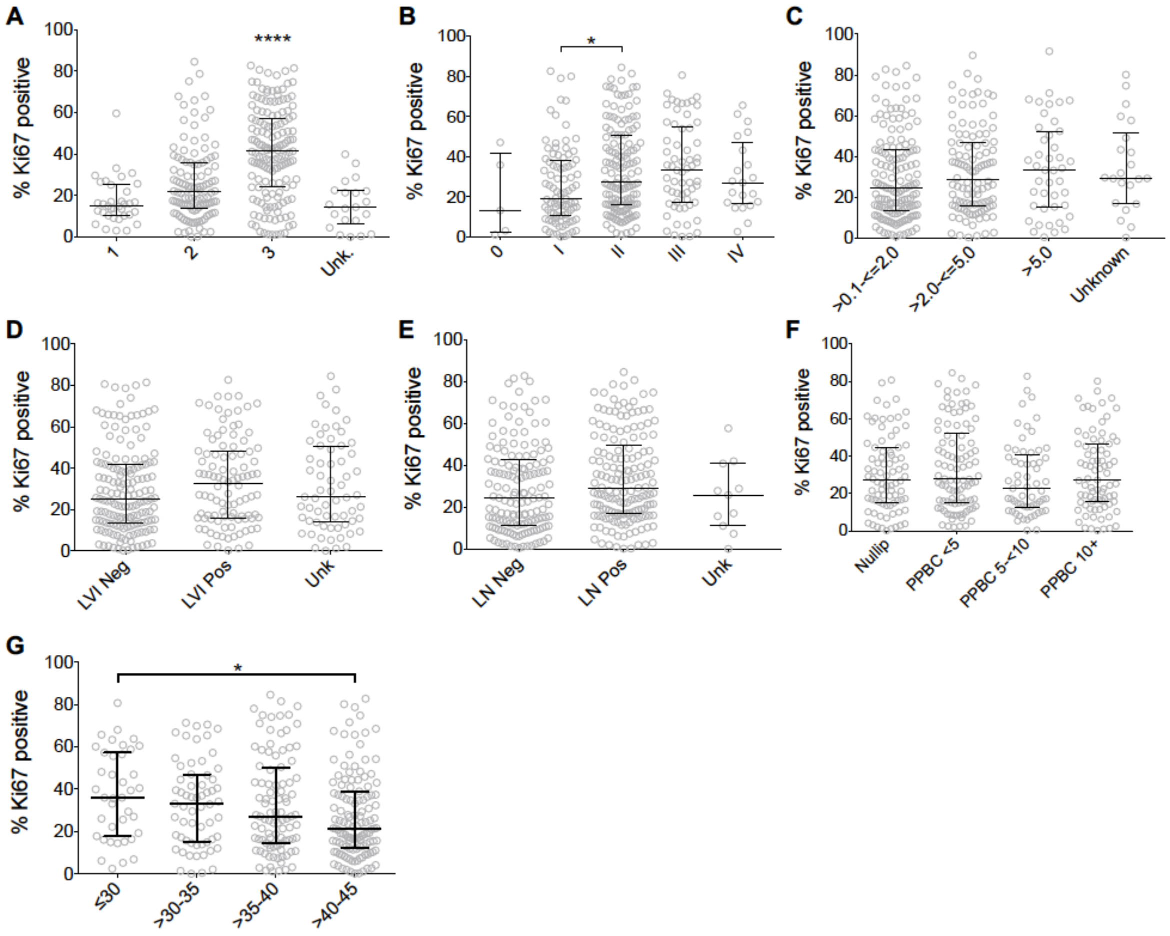

eFigure 6. Intratumoral Ki67 staining index is increased with increasing grade and stage and is reduced with increasing age at diagnosis. Intratumoral Ki67 staining indices by pathological grade **(A)**, clinical stage **(B)**, tumor size **(C)**, lymphovascular invasion (LVI) **(D)**, lymph node (LN) involvement **(E)**, parity status **(F)**, and age at diagnosis **(G)** (One-way ANOVA, Tukey's multiple comparisons test; error bars are median with interquartile range). Nulliparous (Nullip); postpartum breast cancer (PPBC); unknown (unk); positive (Pos); negative (neg). *=p-value<0.05, ****=p-value<0.0001.

© 2019 Goddard ET et al. *JAMA Network Open.*

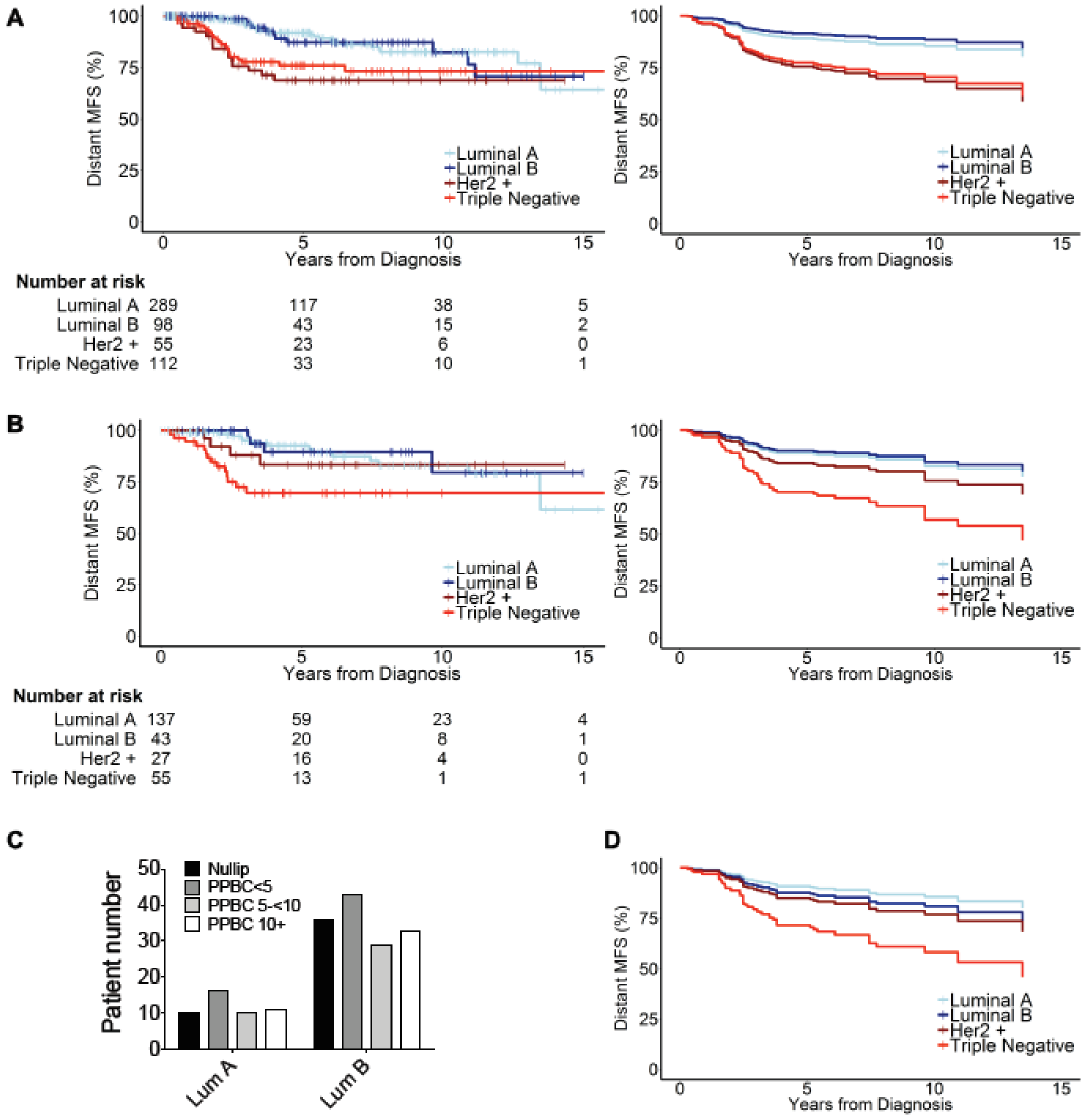

eFigure 7. Distant MFS in the Colorado Young Women's Breast Cancer Cohort by luminal subtype. (A) Distant MFS in the overall Colorado Young Women's Breast Cancer Cohort, categorized by biologic subtype using historic subtype definitions: Luminal A is defined as ER+, PR+, Her2- and luminal B is defined as ER+, PR-, Her2- or ER+, PR+/-, Her2+. Data is unadjusted (left) or adjusted for tumor size, age at diagnosis, and year of diagnosis (right). Luminal A, n=289; luminal B, n=98; Her2+, n=55; Triple negative, n=112. See eTable 4 for statistics. **(B)** Distant MFS in the Ki67 stained subset cohort, categorized by biologic subtype, using the historic Luminal A and B definitions as described in Fig. 4A-B. Data is unadjusted (left) or adjusted for tumor size, age at diagnosis, and year of diagnosis (right). Luminal A, n=137; luminal B, n=43; Her2+, n=27; Triple negative, n=55. See eTable 4 for statistics. **(C)** Number of patients classified as luminal A or luminal B using the Ki67-based definition and categorized by parity group. **(D)** Distant MFS in the Ki67 stained subset cohort, categorized by biologic subtype using the Ki67-based biologic subtype definitions: luminal A is defined as ER+, PR+, Her2-, Ki67 low; luminal B is defined as ER+, PR+/-, Her2+/-, Ki67 high. Data is adjusted for tumor size, age at diagnosis, and year of diagnosis. See eTable 4 for statistics.

© 2019 Goddard ET et al. *JAMA Network Open.*

eTable 1 | University of Colorado metastasis-free survival analysis

UC Cohort Statistical Analysis, Stage I, II, II (N=701)					
	**Log Rank Test P value**	**Univariate P value**	**Hazard Ratio [95% CI]**	**Multivariate P value***	**Hazard Ratio [95% CI]**
**Group**					
**Nulliparous (n=217)**	0.02	Reference	Reference	Reference	Reference
**PPBC<5 (n=175)**		0.009	2.13 [1.21-3.74]	0.16	1.53 [0.85-2.75]
**PPBC 5-<10 (n=153)**		0.006	2.23 [1.26-3.93]	0.02	1.99 [1.10-3.59]
**PPBC 10+ (n=156)**		0.13	1.56 [0.87-2.81]	0.30	1.40 [0.75-2.61]
**UC Cohort Statistical Analysis, Stage I, II patients only (N=550)**					
	**Log Rank Test P value**	**Univariate P value**	**Hazard Ratio [95% CI]**	**Multivariate P value***	**Hazard Ratio [95% CI]**
**Group**					
**Nulliparous (n=185)**	0.001	Reference	Reference	Reference	Reference
**PPBC<5 (n=137)**		0.001	3.69 [1.65-8.25]	0.002	3.51 [1.56-7.89]
**PPBC 5-<10 (n=116)**		<0.001	4.06 [1.81-9.15]	<0.001	5.16 [2.26-11.82]
**PPBC 10+ (n=112)**		0.12	2.02 [0.84-4.89]	0.05	2.57 [1.02-6.48]
**UC Cohort Statistical Analysis, Stage III patients only (N=151)**					
	**Log Rank Test P value**	**Univariate P value**	**Hazard Ratio [95% CI]**	**Multivariate P value***	**Hazard Ratio [95% CI]**
**Group**					
**Nulliparous (n=32)**	0.60	Reference	Reference	Reference	Reference
**PPBC<5 (n=38)**		0.38	0.70 [0.31-1.58]	0.14	0.49 [0.19-1.26]
**PPBC 5-<10 (n=40)**		0.19	0.58 [0.26-1.31]	0.30	0.65 [0.28-1.48]
**PPBC 10+ (n=41)**		0.36	0.69 [0.31-1.52]	0.39	0.69 [0.29-1.61]

*** Multivariable Cox Proportional Hazards regression, adjusted for biologic subtype, age at diagnosis, and year of diagnosis**
95% CI = 95% confidence interval; UC = University of Colorado

© 2019 Goddard ET et al. *JAMA Network Open.*

eTable 2 | University of Colorado metastasis-free survival analysis, stratified by ER status

UC Cohort Statistical Analysis, Stage I, II, III stratified by ER status (N=515)					
	**Log Rank Test P value**	**Univariate P-value**	**Hazard Ratio [95% CI]**	**Multivariate P-value***	**Hazard Ratio [95% CI]**
**Group**					
**Nulliparous, ER+ (n=142)**	<0.001	Reference	Reference	Reference	Reference
**Nulliparous, ER- (n=62)**		0.06	1.95 [0.97-3.93]	0.20	1.60 [0.79-3.26]
**PPBC<10, ER+ (n=218)**		0.12	1.99 [0.84-4.68]	0.19	1.80 [0.75-4.33]
**PPBC<10, ER- (n=93)**		<.001	4.34 [2.12-8.89]	0.004	3.02 [1.44-6.36]

UC Cohort Statistical Analysis, Stage I, II patients only, stratified by ER status (N=410)					
	**Log Rank Test P value**	**Univariate P-value**	**Hazard Ratio [95% CI]**	**Multivariate P-value****	**Hazard Ratio [95% CI]**
**Group**					
**Nulliparous, ER+ (n=125)**	<0.001	Reference	Reference	Reference	Reference
**Nulliparous, ER- (n=49)**		0.02	3.40 [1.26-9.21]	0.008	3.84 [1.42-10.42]
**PPBC<10, ER+ (n=168)**		0.45	1.66 [0.45-6.2]	0.77	1.22 [0.32-4.62]
**PPBC<10, ER- (n=68)**		<0.001	7.07 [2.53-19.82]	<0.001	6.37 [2.26-17.96]

UC Cohort Statistical Analysis, Stage III patients only, stratified by ER status (N=105)					
	**Log Rank Test P value**	**Univariate P-value**	**Hazard Ratio [95% CI]**	**Multivariate P-value****	**Hazard Ratio [95% CI]**
**Group**					
**Nulliparous, ER+ (n=17)**	0.005	Reference	Reference	Reference	Reference
**Nulliparous, ER- (n=13)**		0.34	0.61 [0.22-1.69]	0.19	0.49 [0.17-1.43]
**PPBC<10, ER+ (n=50)**		0.05	3.26 [1.02-10.45]	0.29	2.04 [0.55-7.54]
**PPBC<10, ER- (n=25)**		0.38	1.57 [0.57-4.35]	0.78	1.17 [0.39-3.50]

*** Multivariate logistic regression, adjusted for biologic subtype, stage, age at diagnosis, and year of diagnosis**
**** Multivariate logistic regression, adjusted for biologic subtype, age at diagnosis, and year of diagnosis**
95% CI = 95% confidence interval; UC = University of Colorado; ER = estrogen receptor

© 2019 Goddard ET et al. *JAMA Network Open.*

eTable 3 | Ki67 staining cohort

	Nulliparous (N=72) No. (%)	PPBC <5 years (N=86) No. (%)	PPBC 5-<10 years (N=48) No. (%)	PPBC 10+ (N=66) No. (%)	P-value
**Mean age at diagnosis**	37.2	34.8	39.2	41.5	<0.001[b]
**Biologic subtype**					
Luminal A (ER+, PR+, Her2 neu-)	30 (41.7%)	45 (52.3%)	30 (62.5%)	32 (48.5%)	
Luminal B (ER+, PR+/-, Her2 neu+)	14 (19.4%)	10 (11.6%)	8 (16.7%)	10 (15.2%)	
Her2 neu positive (ER-, PR-)	12 (16.7%)	9 (10.5%)	2 (4.2%)	4 (6.1%)	0.35[a]
Triple negative	14 (19.4%)	20 (23.3%)	7 (14.6%)	16 (24.2%)	
Luminal unknown	2 (2.8%)	2 (2.3%)	1 (2.1%)	4 (6.1%)	
**Updated biologic subtype**					
Luminal A (ER+, PR+/-, Ki67 <14%)	9 (12.5%)	14 (16.3%)	10 (20.83%)	11 (16.7%)	
Lumincal B (ER+, PR+/-, Ki67 ≥14%)	31 (43.1%)	38 (44.2%)	27 (56.3%)	33 (50.0%)	
Her2 neu positive (ER-, PR-)	12 (16.7%)	9 (10.5%)	2 (4.2%)	4 (6.1%)	0.45[a]
Triple negative	14 (19.4%)	20 (23.3%)	7 (14.6%)	16 (24.2%)	
Luminal unknown	6 (8.3%)	5 (5.8%)	2 (4.2%)	2 (3.0%)	
**Estrogen status**					
ER+	46 (63.9%)	57 (66.3%)	39 (81.3%)	46 (69.7%)	0.21[a]
ER-	26.(36.1%)	29 (33.7%)	9 (18.8%)	20 (30.3%)	
**Histologic grade**					
Grade I	6 (8.3%)	10 (11.6%)	10 (20.8%)	6 (9.1%)	
Grade II	23 (31.9%)	29 (33.7%)	15 (31.3%)	30 (45.5%)	0.39[a]
Grade III	40 (55.6%)	42 (48.8%)	22 (45.8%)	27 (40.9%)	
Missing	3 (4.2%)	5 (5.8%)	1 (2.1%)	3 (4.6%)	
**Tumor size**					
0.1—≤2.0 cm	36 (50.0%)	47 (54.7%)	23 (47.9%)	26 (39.4%)	
>2.0—≤5.0 cm	24 (33.3%)	27 (31.4%)	20 (41.7%)	26 (39.4%)	0.28[a]
>5.0 cm	6 (8.3%)	7 (8.14%)	4 (8.3%)	12 (18.2%)	
Missing	6 (8.3%)	5 (5.8%)	1 (2.1%)	2 (3.0%)	
**Stage**					
I	21 (29.2%)	24 (27.9%)	16 (33.3%)	18 (27.3%)	
II	40 (55.6%)	41 (47.7%)	22 (45.8%)	32 (48.5%)	0.82[a]
III	11 (15.3%)	21 (24.42%)	10 (20.8%)	16 (24.2%)	
**Year of Diagnosis**					
1980-2004	27 (37.5%)	25 (29.1%)	15 (31.3%)	34 (51.5%)	0.03[a]
2005-present	45 (62.5%)	61 (70.9%)	33 (68.8%)	32 (48.5%)	
**Mean BMI**	24.6	26.3	26.1	27.3	0.01[b]
**Chemotherapy**					
Yes	48 (66.7%)	59 (68.6%)	34 (70.8%)	41 (62.1%)	
No	5 (6.9%)	7 (8.1%)	5 (10.4%)	6 (9.1%)	0.84[a]
Missing	19 (26.4%)	20 (23.3%)	9 (18.7%)	19 (28.8%)	
**Radiation Therapy**					
Yes	29 (40.3%)	38 (44.2%)	20 (41.7%)	29 (43.9%)	
No	20 (27.8%)	22 (25.6%)	11 (22.9%)	16 (24.2%)	0.99[a]
Missing	23 (32.0%)	26 (30.2%)	17 (35.4%)	21 (31.8%)	
**Patients with Metastasis**	7 (9.7%)	18 (20.9%)	6 (12.5%)	10 (15.2%)	0.24[b]

PPBC, Postpartum Breast Cancer; No., number of patients per group; ER, estrogen receptor; PR, progesterone receptor; DCIS, ductal carcinoma in situ; cm, centimeter; BMI, body mass index

© 2019 Goddard ET et al. *JAMA Network Open.*

eTable 4 | University of Colorado metastasis-free survival analysis, stratified by biologic subtype

UCH cohort using the old definition of luminal A/B (N=554)

Group	Log Rank Test P value	Univariate P value	Hazard Ratio [95% CI]	Multivariate P value*	Hazard Ratio [95% CI]
Luminal A	<0.001	Reference	Reference	Reference	Reference
Luminal B		0.900	1.04 [0.52-2.11]	0.94	1.03 [0.47-2.24]
Her2+		0.001	2.89 [1.54-5.44]	0.008	2.62 [1.28-5.35]
Triple Negative		0.002	2.39 [1.36-4.21]	0.004	2.44 [1.32-4.49]

Ki67 stained subset cohort using the old definition of luminal A/B (N=262)

Group	Log Rank Test P value	Univariate P value	Hazard Ratio [95% CI]	Multivariate P value*	Hazard Ratio [95% CI]
Luminal A	0.003	Reference	Reference	Reference	Reference
Luminal B		0.6700	0.79 [0.26-2.37]	0.72	0.82 [0.26-2.52]
Her2+		0.68	1.26 [0.42-3.81]	0.65	1.30 [0.42-4.03]
Triple Negative		0.002	3.29 [1.57-6.9]	0.004	3.27 [1.47-7.31]

Ki67 stained subset cohort using the updated definition of luminal A/B (N=258)

Group	Log Rank Test P value	Univariate P value	Hazard Ratio [95% CI]	Multivariate P value*	Hazard Ratio [95% CI]
Luminal A	0.007	Reference	Reference	Reference	Reference
Luminal B		0.44	1.51 [0.54-4.22]	0.35	1.71 [0.56-5.28]
Her2+		0.48	1.61 [0.43-6.05]	0.42	1.78 [0.44-7.29]
Triple Negative		0.007	4.21 [1.47-12.05]	0.01	4.42 [1.38-14.17]

***Multivariable Cox Proportional Hazards regression, adjusted for tumor size, age at diagnosis, and year of diagnosis**
95% CI = 95% confidence interval

© 2019 Goddard ET et al. *JAMA Network Open.*
